# Proteomic analysis of proteins responsive to drought stress in barley

**DOI:** 10.1186/s12870-026-08176-8

**Published:** 2026-02-11

**Authors:** Walaa Abdel-Kader Ramadan, Fatma El-Sayed Mahmoud, Mahmoud Hussien Abou-Deif, Mohammed Ali

**Affiliations:** 1 Genetics and Cytology Department, National Research Centre, Dokki, Giza, 12622 Egypt; 2 Genetic Resources Department, Maryout Research Station, Desert Research Center, 1 Mathaf El-Matarya St., El-Matareya, Cairo, 11753 Egypt

**Keywords:** Barley, Drought stress, Polyethylene glycol (PEG), Proteomics, Putative tissue expression, Two dimensional (2-DE) electrophoresis

## Abstract

**Background:**

Drought stress is one of the main environmental factors limiting the development, growth, and crop yield of barley plants. Finding drought-tolerant genes and the proteins they encode that are linked to the interplay between drought tolerance and growth/yield is crucial for enhancing genotypes’ ability to withstand drought and other abiotic stressors. Our study’s objective was to leverage prior proteomic research to identify candidate genes and the proteins they encode that are important in barley’s responses to drought tolerance and to examine how drought stress alters their expression.

**Results:**

In this study we used two-dimensional electrophoresis (2D-gel), mass spectrometry and bioinformatics software to investigate Giza132 barley seedling proteins’ composition and function under drought stress in comparison with control. Our results showed about 56 spots (proteins) from various unique and common proteins, which are related with the ability of barley plants to tolerance. the drought stress. And these proteins have various biological functions and play an important role in drought and other abiotic stress tolerance such as; spot 32 (HORVU4Hr1G089510), spot 47 (HORVU4Hr1G057210) and spot 51 (HORVU4Hr1G016810) which related with L-ascorbate peroxidase enzyme, Alcohol dehydrogenase I enzyme and Fructose-bisphosphate aldolase enzyme, respectively. Moreover, from our results we found the control gel have six unique spots (spots sequence numbers; e.g., 25, 26, 27, 42, 46 and 50), while eight unique spots were detected in the drought stress gel (spots sequence numbers; e.g., 1, 17, 24, 34, 44, 47, 52 and 56). And the remaining 42 spots are common between both gels. And some of these previous proteins are up-regulated and the others are down-regulated expressions under the effect of drought stress. In addition, our data analysis showed that these previous proteins showed various biological functions and some of these functions were related with the ability of drought tolerance in plants. Furthermore, the putative expression patterns of the identified proteins by BAR databases as bioinformatics tools were also completed.

**Conclusions:**

To our knowledge, this is the first proteomic analysis for evaluating the effect of drought stress on the levels of expression of different proteins in Giza132 barley seedling. At the end, this information can be relied upon in future programs related to the production of new barley genotypes that are tolerant to drought and other biotic stresses.

**Supplementary Information:**

The online version contains supplementary material available at 10.1186/s12870-026-08176-8.

## Introduction

One of the most detrimental abiotic stresses for plants is drought, which affects their growth, reproduction, and yield. To adapt to dry conditions, plants use morphological, physiological, biochemical, cellular, and molecular processes [1]. All intracellular processes involve proteins, which are also essential for drought tolerance. As genomics has advanced quickly, proteomics has emerged as a viable technique for identifying proteins resistant to drought that could be employed in marker-assisted selection to improve crops [2]. Protein separation and identification methods, such as two-dimensional gel electrophoresis (2-DE), liquid chromatography, and mass spectrometry (MS) have made tremendous strides in recent years. Database accessibility and searching have also improved [3]. One of the most popular proteomics methods is two-dimensional gel electrophoresis (2-DE), which makes it easy to resolve and view thousands of protein species on a single gel, hence resolving proteoforms [4]. Expression proteomics is used to study the qualitative expression of proteins under different situations [5]. Understanding how distinct genomic regions affect the composition of grain proteins, the function of enzymes, and the expression of particular genes under various growing circumstances is made possible by proteomic [6, 7]. Proteomics in barley is therefore a useful technique for explaining protein expression and how it adds to the grain’s value and drought resistance.

Proteomic technique of agricultural drought response in rice has been revealed by the use of proteomic method in crop plants [7] such as; wheat [8], maize [9], barley [10], soybean [11], bean [12] and sorghum [13]. Specifically, transcriptome and proteome investigations of wheat revealed that the growing grains have a large number of genes that withstand drought [14], and proteins that are crucial for grain development and yield creation in response to drought stress [15, 16]. The combination of functional genomics, proteomics, bioinformatics, breeding, and genetic resources is helping to better understand the genetic and biochemical underpinnings of barley quality features. High throughput screening techniques and breeding programs must include this information in order to combine good yield and agronomic features with good quality [17]. The objective of this study was to investigate the protein composition and function in barley plantlets by the proteomic approach and bioinformatics software. Furthermore, we have examined the alteration in protein expression profiles in early seedling growth of drought tolerance Egyptian barley cultivar (Giza132) as a result of stress circumstances and understand the impact of polyethylene glycol (PEG) osmotic solution as a drought stress on these proteins through classifications of these proteins based on molecular, biological and cellular function, to elucidate the mechanisms behind signaling and tolerance.

## Materials and methods

### Plant material

The Egyptian cultivar of barley (*Hordeum vulgare* L.) used in this study is Giza132 (G132) “drought tolerance” with genetic origin Rihane-05//As46/Aths*2Aths/Lignee686, were kindly obtained by the Barley Research Department, Field Crops Research Institute, Agriculture Research Center (ARC), Giza, Egypt. The research was conducted in the growth champers of the Genetics and Cytology Department, National Research Centre (NRC), Egypt in 2024. Growth chambers conditions are: Light intensities at mid-canopy were maintained at approximately 400 µmols m^− 2^ s^− 1^. A photoperiod of 16 h light and 8 h dark was maintained using a combination of fluorescent lights and incandescent lights. Temperatures were maintained at 23° C daytime and 18° C nighttime and were monitored using chart recorders. Relative humidity was maintained at approximately 50%. The experiment was carried out based on the randomized complete design with three replications. And each replicate consists of one sterilized glass Petri dish with a diameter of 15 cm. Fifteen grains (seeds) were sterilized by immersing in 1% sodium hypochlorite for five minutes, then washed by distilled water three times. After that the sterilized grains were planted on a sterilized glass Petri dish where the filter papers were placed. Eight ml of distilled water was added to each Petri dish. Then, after 24 h 12 ml of the stress solution (10% of polyethylene glycol-6000 (PEG; CAS Number : 25322-68-3, Advent Chembio Pvt. Ltd., Mumbai, India.)) which is equivalent to 0.54 MPa osmotic potential was added to the Petri dishes in compared with control (without PEG ), and petri dishes were incubation for 8th day. All the Petri dishes, water and the seeds bed (Whatman paper) that used in our experiment were sterilized in autoclave [18, 19]. Moreover, based on previous study by Hellal et al., [19], they found a negative relationship between seed germination rate and the concentration of PEG that used, and the results indicate that the optimal concentration for achieving an average germination rate is 10%., so we used this concentration in our current study. For the proteome analysis, three seedlings from each replicate were chosen from the control and drought treatment.

### Protein extraction and two dimensional (2-DE) electrophoresis

Using a mortar and pestle, 0.2 g of three biological replicates from the barley seedling of G132 growth under normal condition (control) and exposed to drought were ground in liquid nitrogen for 2-D electrophoresis. The barley seedling powder was then added to 2 ml of lysis buffer that contained 7 M urea, 2 M thiourea, 4% CHAPS, 18 mM Tris–HCl pH 8.0, and 4% Triton X-100. The powder was mixed with a mixture of protease inhibitors (1 mM PMSF, 0.1 mM pepstatin, 2 mM leupeptin, 1 mM E-64, and 1 mM aprotinin) and 53 u/mL DNase I and 4.9 u/mL RNase. For 20 min, they were incubated at 4 °C. After adding 14 mM of DTT, the samples were centrifuged at 10,000 xg for 20 min at 4 °C until the supernatant was entirely clear and free of lipids. The Bradford technique was used to estimate the protein concentration [20] Employing the BSA-based Bio-Rad Protein Assay. Equal and standard loading quantities were verified on 1-DE gels that were stained with CBB. Protein extracts were diluted for 2-DE analysis using a rehydration solution that contained 1.6% (v/v) DeStreak Reagent (GE Healthcare), 7 M urea, 2 M thiourea, 18 mM Tris–HCl pH 8.0, 4% (w/v) CHAPS, 0.5% (v/v) IPG buffer in the same range as the IPG strip, and 0.002% Bromophenol Blue. Pool samples with 2 mg of protein were placed onto pH 3– 10, 24 cm immobilized pH gradient (IPG) strips (Immobiline DryStrips, GE Healthcare) for the first dimension after being diluted to a final volume of 450 µl. IEF was carried out. at 50 V for 10 h (rehydration), 500 V in gradient for 1 h 30 min, 1000 V in gradient for 1 h 30 min, 2000 V in gradient for 1 h 30 min, 4000 V in gradient for 1 h 30 min, 8000 V in gradient for 2 h and 8000 V holding for 6 h, using Ettan™ IPGphor™ Isoelectric Focusing System (Amersham, Biosciences). IPG strips were equilibrated with 50 mM Tris–HCl (pH 8.8), 6 M urea, 30% (v/v) glycerol, 2% SDS, a trace of Bromophenol Blue, and 10 mg/ml DTT for 15 min prior to the second dimension. This was followed by a second equilibration step using the same buffer, but this time with 25 mg/ml iodoacetamide in place of DTT, for an additional 15 min while being gently shaken. For the second dimension, the focused strips was performed on vertical slabs (20 × 18 × 0.2 cm) where loaded and run on sodium dodecyl sulfate polyacrylamide gel by electrophoresis method SDS-PAGE (13% polyacrylamide) for 30 min, 100 V at room temperature followed by 250 V during 4 h. Proteins were visualized with silver stain dyes [21]. An ImageScanner desktop device (Amersham, Biosciences) was used to scan the 2-DE gels. Images were obtained using the LabScan scanning application, in transmission mode, at a greyscale level of 16 bips, at 300 dpi, with a magnification factor of 1:1 (100%) and saved as TIFF (Tag Image File Format) files. The ImageMasterTM 2-D Platinum 5.0 software (Amersham, Biosciences) was used to analyze the images.

### Image analysis and protein identification

The proteins (spots) in gel images were detected and quantified using the default settings of the RegStatGel software, ImageJ and Melanie v.9 software. And the detected spots on the gel were then contrasted with the NCBI and Swiss-Prot databases and an exact match was found through the Mascot search program [7].Furthermore, the Swiss-Prot, Phytazome 13, and NCBI protein databases were examined for the annotation of the detected proteins in their descriptions. Moreover, the search results filtered based on their probability if it was ≥ 95 and 99% and consisted of two or more identified peptides, the peptide and protein identifications were accepted. The false discovery rate (FDR) threshold was set at 0.01 (strict), and the target FDR (relaxed) was 0.05 using the percolator node. In addition, we select the spots with highly percentage of coverage (Coverage %). Numbers of unique and common spots detected in 2-DE analysis in the control and subjected to drought stress were analysis.

### Identification and functional assignments for protein-associated with dots in *H. vulgare*

Putative sequences of protein from dots analysis were used as a query to search against the *H. vulgare* genomics that we already get from NCBI genomics (https://phytozome-next.jgi.doe.gov/blast-search, accessed on 25 Dec.2023). The alignment sequence was then examined with the Phytozome and KEGG databases in order to predict the biological function of these chosen candidate proteins that were linked to different *H. vulgare* dots. Ultimately, we obtain 53 genes that are closely associated with different functions in *H. vulgare* [22–24].

### Putative expression pattern of our target genes at transverse and sagittal sections based on *H. vulgare*transcript expression

Profiles of putative expression of 47 genes from *H. vulgare* were extracted based on *H. vulgare* transcript expression database from three tissues of transverse and sagittal sections at different times, including; 4D- SMT, 4D-END, 4D-EMB, 8D- SMT, 8D-END, 8D-EMB, 16D- SMT, 16D-END, 16D-EMB, 24D- SMT, 24D-END, 24D-EMB, 32D- SMT, 32D-END and 32D-EMB (see supplementary Table 1). The creation of expression profiles was done using the barley plant Electronic Fluorescent Pictograph Browsers (barley eFP browsers) (http://bar.utoronto.ca/eplant_Arabidopsis/) accessed on 25 April 2024 [25–30].

### Putative expression pattern of our target genes at various tissue, whole spike and provascular Tissue based on *H. vulgare*transcript expression

The putative expression profiles of 47 genes from *H. vulgare* were extracted based on *H. vulgare* transcript expression database from various tissues, whole spike and provascular tissue such as; Vegatative Apex (I-Va-SAM), Double Ridhe (I-Dr-IM), Double Ridhe (I-Dr-LRM), Double Ridhe (I-Dr-SRM), Triple Mound (I-Tm-IM), Triple Mound (I-Tm-LSM), Triple Mound (I-Tm-CSM), Glume Primordium (I-Gp-IM), Glume Primordium (I-Gp-LSM), Glume Primordium (I-Gp-CSM), Legmma Primodium (I-Lp-IM), Legmma Primodium (I-Lp-LSM), Legmma Primodium (I-Lp-CSM), Stamen Primordium (I-Sp-IM), Stamen Primordium (I-Sp-LSM), Stamen Primordium (I-Sp-CSM), Awa Primordium (I-Ap-IM), Awa Primordium (I-Ap-LSM), Awa Primordium (I-Ap-CSM), White Anther (I-Wa-IM), Leaf Blade Base (I-LBB), Root Apical Meristem (I-RAM), Double Ridge (W-DR), Triple Mound (W-TM), Glume Primordium (W-GP), Lemma Primordium (W-LP), Stamen (W-SP), Awn Primordium (W-AP), Double Ridge (R-DR), Triple Mound (R-GP), Glume Primordium (R-GP), Lemma Primordium (R-LP), Stamen (R-SP) and Awn Primordium (R-AP) (see supplementary Table 1. The Electronic Fluorescent Pictograph Browsers for barley plants were used to develop expression profiles. (barley eFP browsers) (http://bar.utoronto.ca/eplant_Arabidopsis/) accessed on 30April 2024 [25–30].

### Putative expression pattern of our target genes at various tissues under light and shade based on *H. vulgare*transcript expression

Many researchers have been studied the impact of light and dark on leaf respiration under drought stress, and their results referee to epistatic relationship between the leaf respiration light and dark in one side and the leaf carbon metabolism, N- and P-based rates and CO2 concentration from the other side [31, 32]. So, we analyses the putative expression profiles of 47 genes from *H. vulgare* that were extracted based on *H. vulgare* transcript expression database from various tissues under light and shade conditions, including; Double Ridhe (I-Dr-IM), Double Ridhe (I-Dr-LRM), Double Ridhe (I-Dr-SRM), Triple Mound (I-Tm-IM), Triple Mound (I-Tm-LSM), Triple Mound (I-Tm-CSM), Glume Primordium (I-Gp-IM), Glume Primordium (I-Gp-LSM), Glume Primordium (I-Gp-CSM), Legmma Primodium (I-Lp-IM), Legmma Primodium (I-Lp-LSM), Legmma Primodium (I-Lp-CSM), Stamen Primordium (I-Sp-IM), Stamen Primordium (I-Sp-LSM), Stamen Primordium (I-Sp-CSM), Awa Primordium (I-Ap-IM), Awa Primordium (I-Ap-LSM) and Awa Primordium (I-Ap-CSM), (see supplementary Table S3). Expression profiles were created using the barley plant Electronic Fluorescent Pictograph Browsers (barley eFP browsers) (http://bar.utoronto.ca/eplant_Arabidopsis/) accessed on 5 June 2024 [25–30].

## Results

### Protein extraction and identification in *H. vulgare* leaves

**Fig. 1:**
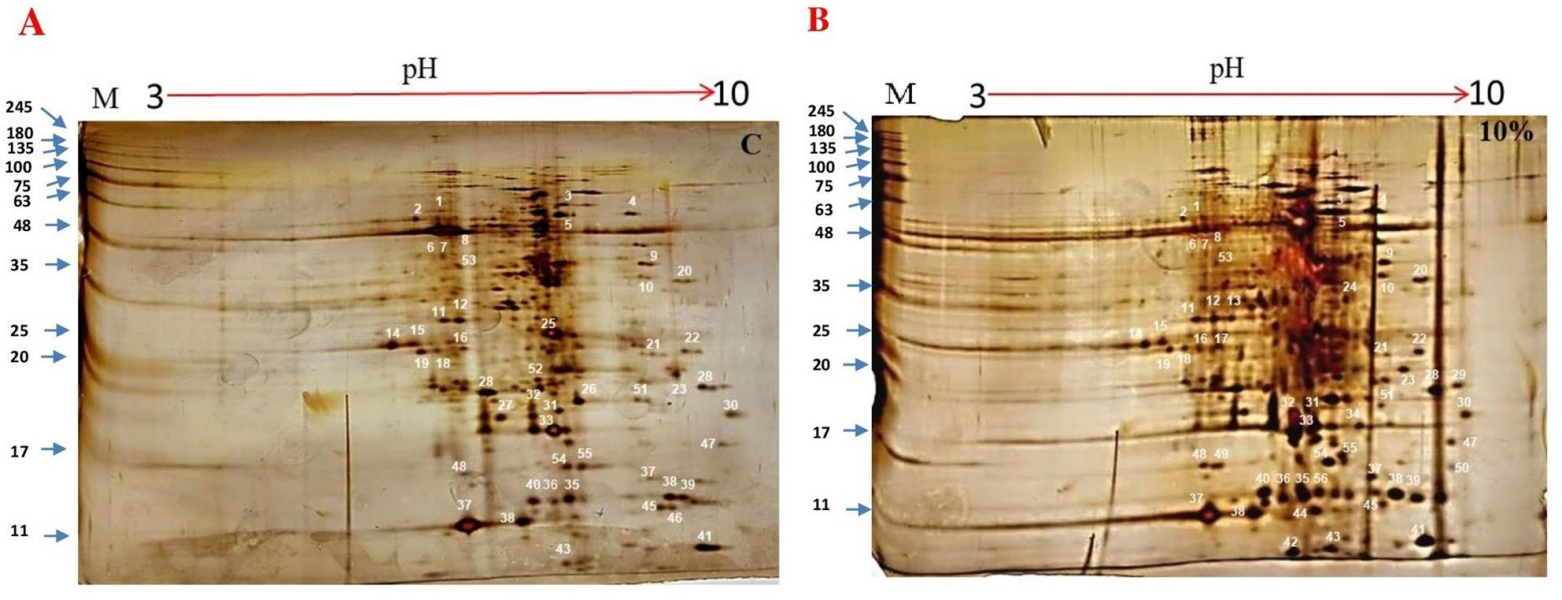
Representative two-dimensional gel electrophoresis (2-DE) maps of barley leaf proteins in Giza1132 (G132). The proteins were isolated from the pool of three biological replicates from the barley seedling of G132 growth under normal condition (control) (**A**) and exposed to drought (**B**). The spots were visualized by silver staining. Differentially accumulated protein spots are indicated by white numbers. Spots indicate the differentially expressed protein spots whose expression levels were significantly induced or down regulated in G132. Protein Marker starts from 245 to 11 KDa

Leaf proteins were taken out of barley leaf under drought stress and without drought stress (control), and then the proteins were separated in the 2D gels using electrophoreticism. After that, using 2-DE, we examined the protein patterns in the pH range of 3–10. The barley proteins that were visible were packed, and all our spots (dots) became visible in the pH range of 5–10. Moreover, we used 2-DE to further examine the protein patterns of these areas and the resulting 2DE images of the proteins in barley leaves are displayed in Fig. 1A, B and Supplementary Fig. 1. After being chosen from 2-D gels, spots were analyzed using a MASCOT database. These protein spots were located through a search in barley, rice, wheat and other cereal crops protein sequences from Swiss-Prot, phytozome 13 and NCBI databases and the proteins with highly percentage of coverage (Coverage %) were selected. As results, 56 spots (barely proteins) were chosen for identification, according to the putative functions of each spot, see supplementary Table 1. Our results were filtered based on the probability, and when the probability was ≥ 95 and 99% and consisted of two or more identified peptides, the peptide and protein (Spots) identifications were accepted. The false discovery rate (FDR) threshold was set at 0.01 (strict), and the target FDR (relaxed) was 0.05 using the percolator node. Furthermore, from our results we found the control gel have six unique spots (spots sequence numbers; e.g., 25, 26, 27, 42, 46 and 50), while eight unique spots were detected in the drought stress gel (spots sequence numbers e.g., 1, 17, 24, 34, 44, 47, 52 and 56). And the remaining 42 spots are common between both gels see see supplementary Table 1.

### Functional annotations of the Gene-associated dots in *H. vulgare*

In order to estimate the possible activities of our target genes, the *H. vulgare* genomic sequence served as a template for searching the protein dot sequence. We discovered 53 genes that may be involved in barley’s resistance to drought and other abiotic stressors based on sequence homology (see Table 1). Subsequently, multiple databases, including the Phytozome, NCBI, InterPro, and KEGG databases, predicted additional function annotations for these genes. Contextually, these genes were linked to numerous biological processes, such as HORVU.MOREX.r3.2HG0202350 (gi|251832986), HORVU.MOREX.r3.2HG0202350 (gi|121340), HORVU.MOREX.r3.UnG0798500 (gi|108711272), HORVU.MOREX.r3.UnG0808370 (gi|110915608), HORVU.MOREX.r3.5HG0483130 (RBL_AGRST), HORVU.MOREX.r3.5HG0490180 (gi|32765549), HORVU.MOREX.r3.1HG0001110 (gi|17425184), HORVU.MOREX.r3.1HG0001420 (gi|53854906), HORVU.MOREX.r3.2HG0167220 (BAS1_WHEAT), HORVU5Hr1G124160 (CAA32900.1), HORVU.MOREX.r3.2HG0120840 (gi|326496957), HORVU.MOREX.r3.6HG0591100 (CAA36498.1), HORVU.MOREX.r3.2HG0138840 (XP_002518555.1), HORVU.MOREX.r3.3HG0276570 (gi|326493350), HORVU.MOREX.r3.5HG0421460 (gi|326493798), HORVU.MOREX.r3.5HG0511770 (AAF98561.1), HORVU.MOREX.r3.4HG0417260 (AAC67246.1), HORVU.MOREX.r3.5HG0421370 (gi|326493636), HORVU.MOREX.r3.4HG0385790 (CAA06996.1), HORVU.MOREX.r3.7HG0720010 (GI:18412879), HORVU.MOREX.r3.4HG0345760 (AAO42684.1), HORVU.MOREX.r3.3HG0304680 (ACO44685.1) and HORVU.MOREX.r3.4HG0392240 (CAA38036.1) that associated with Plastid glutamine synthetase 2, Glutamine synthetase leaf isozyme, Chloroplastic, ATP synthase beta chain, putative, ATP synthase beta subunit, Ribulose bisphosphate carboxylase large chain, Predicted: 20 kDa chaperonin, chloroplastic-like respectivelly, Cytosolic heat shock protein 90, LMW- glutenin subunit group 3 type II, LMW- glutenin, 2-cys peroxiredoxin BAS1, Chlorophyll a-b binding protein 1, chloroplastic, thioredoxin peroxidase, Elongation factor Tu, chloroplastic, cysteine-rich receptor-like protein kinase 10, isocitrate dehydrogenase, glycine rich protein, RNA binding protein, UDP-glucose 6-dehydrogenase, Beta-amylase, enolase, L-ascorbate peroxidase, Leucine-rich repeat family protein LRR, Alcohol dehydrogenase I, fructose-bisphosphate aldolase and Heat shock protein 21, chloroplast, respectively (see Table 1).

**Table 1:** Barley proteins identified and catalogued from the 2-DE

Spot No.	NCBI identifier	Protein name (organism)	Hordeum vulgare r1 ID	Hordeum vulgare Morex V3	E.C	InterPro	Chromosome Location
1	gi|251,832,986	Plastid glutamine synthetase 2 [*T. Aestivum*]	HORVU2Hr1G111300	HORVU.MOREX.r3.2HG0202350	6.3.1.2	IPR008146, IPR008147, IPR014746	chr2H:722462607.722470196 reverse
2	gi|121,340	Glutamine synthetase leaf isozyme, Chloroplastic	HORVU6Hr1G074030	HORVU.MOREX.r3.6HG0613270	6.3.1.2	IPR008146, IPR008147, IPR014746	chr6H:495214922.495218750 reverse
3	gi|108,711,272	ATP synthase beta chain, putative [*O. sativa* Japonica Group]	HORVU7Hr1G088200	HORVU.MOREX.r3.UnG0798500	3.6.3.14	IPR000793, IPR020546, IPR027417, IPR020547, IPR001469, IPR000194, IPR024034	chr7H:533883311.533885366 forward
4	gi|110,915,608	ATP synthase beta subunit [*Calotheca brizoides*]	HORVU6Hr1G049250	HORVU.MOREX.r3.UnG0808370	3.6.3.14	IPR000793, IPR004100, IPR005722, IPR027417, IPR024034, IPR003593, IPR000194,	chr6H:299512162.299514069 reverse
5	RBL_AGRST	Ribulose bisphosphate carboxylase large chain Agrostis stolonifera	HORVU7Hr1G088190	HORVU.MOREX.r3.5HG0483130	6.3.1.2	IPR000685, IPR017443	chr7H:533880816.533882550 reverse
6	gi|357,158,586	Predicted: 20 kDa chaperonin, chloroplastic-like (*B. distachyon*)	HORVU5Hr1G062310	HORVU.MOREX.r3.5HG0480620		IPR020818, IPR011032, IPR017416	chr5H:485025059.485029334 reverse
7	gi|51,090,748	Putative chaperonnin 21 precursor [*O. sativa* Japonica]	HORVU7Hr1G033900	HORVU.MOREX.r3.7HG0661950		IPR020818, IPR011032, IPR017416	chr7H:70159514.70163687 forward
8	gi|32,765,549	Cytosolic heat shock protein 90 [*H. vulgare*]	HORVU5Hr1G072420	HORVU.MOREX.r3.5HG0490180		IPR001404, IPR020575, IPR020568, IPR013320, IPR003594	chr5H:535441664.535446369 reverse
9	gi|326,516,152	Predicted protein (*H. vulgare*)	HORVU3Hr1G015680	HORVU.MOREX.r3.3HG0232890	1.1.1.54	IPR011032, IPR020843, IPR016040, IPR013149, IPR002085	chr3H:36873414.36875746 forward
10	gi|326,497,973	Predicted protein (*H. vulgare*)	HORVU3Hr1G015640	HORVU.MOREX.r3.3HG0232910	1.1.1.54	IPR011032, IPR020843, IPR016040, IPR013149, IPR002085	chr3H:36886533.36888603 reverse
11	gi|17,425,184	LMW- glutenin subunit group 3 type II	HORVU1Hr1G001020	HORVU.MOREX.r3.1HG0001110		IPR001954, IPR013771, IPR016140, IPR001376	chr1H:2524727.2525648 forward
12	gi|121,451	Glutenin, high molecular weight subunit PC237	HORVU1Hr1G000990	HORVU.MOREX.r3.1HG0001420		IPR001954, IPR013771, IPR016140, IPR001376	chr1H:2511291.2816446 reverse
13	gi|53,854,906	LMW- glutenin	HORVU1Hr1G001420	HORVU.MOREX.r3.1HG0001420		IPR001954, IPR013771, IPR016140, IPR001376	chr1H:3252713.3253756 forward
14	BAS1_WHEAT	2-cys peroxiredoxin BAS1 (*T. aestivum*)	HORVU2Hr1G073760	HORVU.MOREX.r3.2HG0167220	1.11.1.15	IPR019479, IPR012336, IPR013740	chr2H:532288069.532292322 reverse
15	BAS1_WHEAT	2-cys peroxiredoxin BAS1 (*T. aestivum*)	HORVU2Hr1G073760	HORVU.MOREX.r3.2HG0167220	1.11.1.15	IPR019479, IPR012336, IPR013740	chr2H:532288069.532292322 reverse
16	BAS1_WHEAT	2-cys peroxiredoxin BAS1 (*T. aestivum*)	HORVU2Hr1G073760	HORVU.MOREX.r3.2HG0167220	1.11.1.15	IPR019479, IPR012336, IPR013740	chr2H:532288069.532292322 reverse
17	BAS1_WHEAT	2-cys peroxiredoxin BAS1 (*T. aestivum*)	HORVU2Hr1G073760	HORVU.MOREX.r3.2HG0167220	1.11.1.15	IPR019479, IPR012336, IPR013740	chr2H:532288069.532292322 reverse
18	BAS1_WHEAT	2-cys peroxiredoxin BAS1 (*T. aestivum*)	HORVU2Hr1G073760	HORVU.MOREX.r3.2HG0167220	1.11.1.15	IPR019479, IPR012336, IPR013740	chr2H:532288069.532292322 reverse
19	BAS1_WHEAT	2-cys peroxiredoxin BAS1 (*T. aestivum*)	HORVU2Hr1G073760	HORVU.MOREX.r3.2HG0167220	1.11.1.15	IPR019479, IPR012336, IPR013740	chr2H:532288069.532292322 reverse
20	BAS1_WHEAT	2-cys peroxiredoxin BAS1(*T. aestivum*)	HORVU2Hr1G073760	HORVU.MOREX.r3.2HG0167220	1.11.1.15	IPR019479, IPR012336, IPR013740	chr2H:532288069.532292322 reverse
21	gi|110,915,608	ATP synthase beta subunit [*C. brizoides*]	HORVU6Hr1G049250	HORVU.MOREX.r3.UnG0808370	3.6.3.14	IPR000793, IPR004100, IPR005722, IPR027417, IPR024034, IPR003593, IPR000194,	chr6H:299512162.299514069 reverse
22	RBL_AGRST	Ribulose bisphosphate carboxylase large chain Agrostis stolonifera	HORVU7Hr1G088190	HORVU.MOREX.r3.5HG0483130	6.3.1.2	IPR000685, IPR017443	chr7H:533880816.533882550 reverse
23	CAA32900.1	Chlorophyll a-b binding protein 1, chloroplastic	HORVU5Hr1G124160	HORVU5Hr1G124160			
24	gi|326,496,957	Thioredoxin peroxidase (*H. vulgare*)	HORVU2Hr1G026810	HORVU.MOREX.r3.2HG0120840	1.11.1.15	IPR019479, IPR024706, IPR013740, IPR012336	chr2H:86326177.86327145 reverse
25	CAA36498.1	Elongation factor Tu, chloroplastic (*A.thaliana*)	HORVU6Hr1G053680	HORVU.MOREX.r3.6HG0591100	3.6.5.3	IPR004541, IPR004160, IPR004161, IPR009001, IPR027417, IPR000795, IPR009000	chr6H:336377046.336380003 reverse
26	XP_002518555.1	Cysteine-rich receptor-like protein kinase 10 (*R.communis*)	HORVU2Hr1G044650	HORVU.MOREX.r3.2HG0138840	2.7.11.1	IPR000719, IPR002290, IPR002902, IPR001245, IPR013320, IPR011009	chr2H:232982051.232985925 reverse
27	gi|326,493,350	Isocitrate dehydrogenase (*H. vulgare*)	HORVU3Hr1G059060	HORVU.MOREX.r3.3HG0276570	1.1.1.42	IPR004790, IPR024084	chr3H:446058317.446062780 reverse
28	gi|326,493,798	Glycine rich protein, RNA binding protein (*H. vulgare*)	HORVU5Hr1G002150	HORVU.MOREX.r3.5HG0421460		IPR000504, IPR012677	chr5H:6152068.6153106 reverse
29	AAF98561.1	UDP-glucose 6-dehydrogenase (*Z. mays*)	HORVU5Hr1G096370	HORVU.MOREX.r3.5HG0511770	1.1.1.22	IPR001732, IPR006115, IPR008927, IPR014027, IPR016040, IPR014026, IPR017476	chr5H:601141343.601145787 reverse
30	CAE02614.1	Protein RAFTIN 1B (*T.aestivum*)	HORVU7Hr1G048820	HORVU.MOREX.r3.7HG0677930		IPR004873	chr7H:167861952.167864773 reverse
31	CAB40384.1	Oxygen-evolving enhancer protein 3 − 1, chloroplastic	HORVU0Hr1G003270	HORVU.MOREX.r3.2HG0173200		IPR023222,IPR008797	chrUn:13485181.13486545 forward
32	AAC67246.1	Beta-amylase	HORVU4Hr1G089510	HORVU.MOREX.r3.4HG0417260	3.2.1.2	IPR001554, IPR001371, IPR017853, IPR013781	chr4H:642560529.642564723 reverse
33	CAA32900.1	Chlorophyll a-b binding protein 1, chloroplastic	HORVU5Hr1G124160	HORVU5Hr1G124160		IPR022796, IPR001344, IPR023329	chr5H:665427412.665428656 forward
34	RBS3_WHEAT	Ribulose-bisphosphate carboxylase small chain	HORVU5Hr1G038630	HORVU.MOREX.r3.5HG0469020	4.1.1.39	IPR024680, IPR000894, IPR024681	chr5H:277205685.277206539 reverse
35	RBS3_WHEAT	Ribulose-bisphosphate carboxylase small chain	HORVU0Hr1G024560	HORVU.MOREX.r3.5HG0468970	4.1.1.39	IPR024680, IPR000894, IPR024681	chrUn:127763210.127837011 forward
36	RBS3_WHEAT	Ribulose-bisphosphate carboxylase small chain	HORVU0Hr1G026090	HORVU.MOREX.r3.1HG0037500	4.1.1.39	IPR024680, IPR000894, IPR024681	chrUn:137434093.137434496 forward
37	gi|326,505,132	Predicted protein (*H. vulgare*)	HORVU7Hr1G001030	HORVU.MOREX.r3.7HG0635350		IPR002035	chr7H:2241184.2347181 forward
38	KAE8821856	70 kDa heat shock protein (*H. vulgare*)	HORVU5Hr1G032650	HORVU.MOREX.r3.5HG0451540		IPR012725, IPR029048, IPR013126, IPR029047	chr5H:214877902.214888753 reverse
39	gi|326,493,798	Glycine rich protein, RNA binding protein (*H. vulgare*)	HORVU5Hr1G002150	HORVU.MOREX.r3.5HG0421460		IPR000504, IPR012677	chr5H:6152068.6153106 reverse
40	gi|326,493,798	Glycine rich protein, RNA binding protein (*H. vulgare*)	HORVU5Hr1G002150	HORVU.MOREX.r3.5HG0421460		IPR000504, IPR012677	chr5H:6152068.6153106 reverse
41	gi|326,493,636	Enolase (*H. vulgare*)	HORVU5Hr1G002040	HORVU.MOREX.r3.5HG0421370	4.2.1.11	IPR000941, IPR029065, IPR029017, IPR020810, IPR020811	chr5H:5791249.5802408 reverse
42	GI:9,955,530	Putative hydrolase (*A. thaliana*)	HORVU2Hr1G029840	HORVU.MOREX.r3.2HG0123830	3.1.1.3	IPR029059, IPR029058, IPR000639, IPR000073	chr2H:108258493.108263191 reverse
43	GI:27,545,490	S-receptor kinase 13–18 (*A. lyrata*)	HORVU4Hr1G004890	HORVU.MOREX.r3.4HG0335040	2.7.11.1	IPR001480, IPR003609, IPR001245, IPR013320, IPR024171, IPR011009, IPR000719	chr4H:10704209.10708712 reverse
44	AAF02858.1	Proteasome subunit alpha type-5-A (*A. thaliana*)	HORVU4Hr1G040770	HORVU.MOREX.r3.5HG0456950	3.4.25.1	IPR000426, IPR029055, IPR001353, IPR023332	chr4H:315062727.315067562 reverse
45	AAC32060.1	20 S proteasome subunit PAE1 (*A. thaliana*)	HORVU5Hr1G038710	HORVU.MOREX.r3.4HG0370860	3.4.25.1	IPR029055, IPR000426, IPR001353, IPR023332	chr5H:278441769.278446391 reverse
46	GI:223,452,304	Protein-serine/threonine kinase (*G. max*)	HORVU7Hr1G043150	HORVU.MOREX.r3.7HG0671890	2.7.11.1	IPR000719, IPR002290, IPR001245, IPR013320, IPR011009	chr7H:128676716.128679099 reverse
47	CAA06996.1	L-ascorbate peroxidase (*H. vulgare)*	HORVU4Hr1G057210	HORVU.MOREX.r3.4HG0385790	1.11.1.11	IPR010255, IPR000823, IPR002207, IPR002016	chr4H:480951275.480954017 reverse
48	ADK56176.1	Glycosyltransferase 75 (*T. aestivum*)	HORVU4Hr1G038960	HORVU.MOREX.r3.4HG0369570	5.4.99.30	IPR004901, IPR029044	chr4H:297691097.297693678 forward
49	CAA77237.1	Reversibly glycosylated polypeptide (*T.aestivum*)	HORVU3Hr1G073780	HORVU.MOREX.r3.3HG0290880	5.4.99.30	IPR004901, IPR029044	chr3H:555352768.555355130 reverse
50	GI:18,412,879	Leucine-rich repeat family protein LRR (*A. thaliana*)	HORVU7Hr1G090240	HORVU.MOREX.r3.7HG0720010		IPR006553	chr7H:548933249.548935805 reverse
51	AAO42684.1	Alcohol dehydrogenase I (*O. eichingeri*)	HORVU4Hr1G016810	HORVU.MOREX.r3.4HG0345760	1.1.1.1	IPR011032, IPR016040, IPR013154, IPR013149, IPR002085	chr4H:70912803.70929994 forward
52	ACO44685.1	Fructose-bisphosphate aldolase (*H. vulgare*)	HORVU3Hr1G088540	HORVU.MOREX.r3.3HG0304680	4.1.2.13	IPR029769, IPR013785, IPR000741	chr3H:625238589.625240962 forward
53	CAA38036.1	Heat shock protein 21, chloroplastic	HORVU4Hr1G063350	HORVU.MOREX.r3.4HG0392240		IPR031107, IPR002068, IPR008978	chr4H:530910254.530911640 forward
54	CAA38036.1	Heat shock protein 21, chloroplastic	HORVU4Hr1G063350	HORVU.MOREX.r3.4HG0392240		IPR031107, IPR002068, IPR008978	chr4H:530910254.530911640 forward
55	CAA38036.1	Heat shock protein 21, chloroplastic	HORVU4Hr1G063350	HORVU.MOREX.r3.4HG0392240		IPR031107, IPR002068, IPR008978	chr4H:530910254.530911640 forward
56	CAA38036.1	Heat shock protein 21, chloroplastic	HORVU4Hr1G063350	HORVU.MOREX.r3.4HG0392240		IPR031107, IPR002068, IPR008978	chr4H:530910254.530911640 forward

On the other hand, the gel analysis for control sample has five unique spots such as; spot 25 (HORVU6Hr1G053680), spot 26 (HORVU2Hr1G044650), spot 27 (HORVU3Hr1G059060), spot 28 (HORVU5Hr1G002150) and spot 46 (HORVU7Hr1G043150) and these previous spots were related to various proteins such as; Elongation factor Tu, chloroplastic (*A.thaliana*), Cysteine-rich receptor-like protein kinase 10 (*Ricinus communis*), Isocitrate dehydrogenase (*H. vulgare*), Glycine rich protein, RNA binding protein (*H. vulgare*) and Protein-serine/threonine kinase (*Glycine max*), respectively (Fig. 1; Table 1). Additionally, after analysis the two gels from leaf sample under 10% PEG and control we have found 26 common spots and most of these spots were significantly increased in the size under the effect of stress in compared with the control, which mean the drought stress has positively effect on the expression on these proteins (shown in Fig. 1). These common proteins such as; spot 1 (HORVU2Hr1G111300), spot 2 (HORVU6Hr1G074030), spot 3 (HORVU7Hr1G088200), spot 6 (HORVU5Hr1G062310), spot 7 (HORVU7Hr1G033900), spot 8 (HORVU5Hr1G072420), spot 11 (HORVU1Hr1G001020), spot 12 (HORVU1Hr1G000990), spots 14, 15, 16, 18, 19 (HORVU2Hr1G073760), spot 21 (HORVU6Hr1G049250), spot 23 and 33 (HORVU5Hr1G124160), spot 30 (HORVU7Hr1G048820), spot 31 (HORVU0Hr1G003270), spot 32 (HORVU4Hr1G089510), spot 47 (HORVU4Hr1G057210), spot 48 (HORVU4Hr1G038960), spot 51(HORVU4Hr1G016810), spot 52 (HORVU3Hr1G088540), and spots 53, 54, 55 (HORVU4Hr1G063350) see Fig. 1; Table 1. Furthermore, these previous common spots are related with various proteins such as; Plastid glutamine syntheses 2 [*T. Aestivum*], Glutamine synthetase leaf isozyme, Chloroplast, ATP synthase beta chain, putative [*O. sativa* Japonica Group], Predicted: 20 kDa chaperon, chloroplast-like (*B. distachyon*), Putative chaperon 21 precursor [*O. sativa* Japonica], Cytosolic heat shock protein 90 [*H. vulgare*], Glutenin, LMW- glutenin subunit group 3 type II, high molecular weight subunit PC237, 2-cys peroxiredoxin BAS1 (*T. aestivum*), ATP synthase beta subunit [*Calotheca brizoides*], Chlorophyll a-b binding protein 1, chloroplast, Protein RAFTIN 1B (*T. aestivum*), Oxygen-evolving enhancer protein 3 − 1, chloroplastic, Beta-amylase, L-ascorbate peroxidase (*H. vulgare*), Glycosyltransferase 75 (*T. aestivum*), Alcohol dehydrogenase I (*O. eichingeri*), Fructose-bisphosphate aldolase (*H. vulgare*) and Heat shock protein 21, chloroplast, respectively. And from our data analysis we found these previous proteins showed various biological functions and some of these functions were related with the ability of drought tolerance in plants see Table 1.

### The putative expression of our target genes at different transverse and sagittal sections based on*H. vulgare*transcript expression using barley eFP browsers tool

**Fig. 2:**
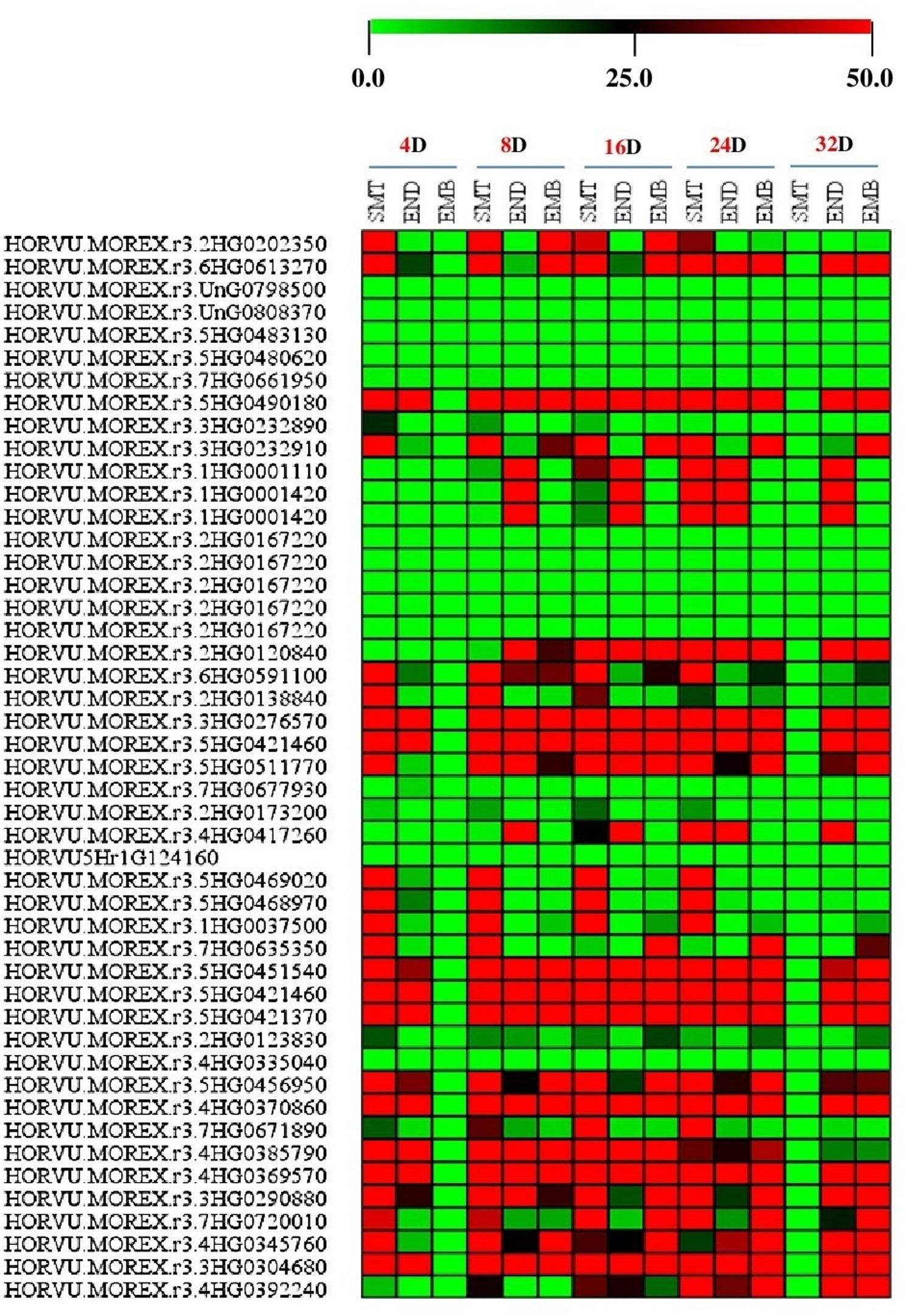
Heat map represent the putative expression of our target genes at various transverse and sagittal sections based on *H. vulgare* transcript expression using barley eFP browsers tool. The colour represents the expression scale (the more intense the red color, the more gene expression). Multi Experiment Viewer (MeV_4_9_0) software was used to depict transcript levels

Based on the transcript expression of *H. vulgare*, the potential expression of our target genes in various transverse and sagittal sections was predicted using the Barley eFP browsers tool. The findings demonstrated that all fifteen tissues had high expression levels of the majority of our target genes from *H. vulgare*. (e.g. 4D- SMT, 4D-END, 4D-EMB, 8D- SMT, 8D-END, 8D-EMB, 16D- SMT, 16D-END, 16D-EMB, 24D- SMT, 24D-END, 24D-EMB, 32D- SMT, 32D-END and 32D-EMB) see Fig. 2 For example, HORVU.MOREX.r3.2HG0202350 (gi|251832986), HORVU.MOREX.r3.6HG0613270 (gi|121340), HORVU.MOREX.r3.5HG0490180 (gi|32765549), HORVU.MOREX.r3.3HG0232910 (gi|326497973), HORVU.MOREX.r3.2HG0120840 (gi|326496957), HORVU.MOREX.r3.6HG0591100 (CAA36498.1), HORVU.MOREX.r3.2HG0138840 (XP_002518555.1), HORVU.MOREX.r3.3HG0276570 (gi|326493350), HORVU.MOREX.r3.5HG0421460 (gi|326493798), HORVU.MOREX.r3.5HG0511770 (AAF98561.1), HORVU.MOREX.r3.4HG0417260 (AAC67246.1), HORVU.MOREX.r3.5HG0451540 (KAE8821856), HORVU.MOREX.r3.5HG0421460 (gi|326493798), HORVU.MOREX.r3.4HG0385790 (CAA06996.1), HORVU.MOREX.r3.4HG0369570 (ADK56176.1), HORVU.MOREX.r3.3HG0290880 (CAA77237.1), HORVU.MOREX.r3.7HG0720010 (GI:18412879), HORVU.MOREX.r3.4HG0345760 (AAO42684.1), HORVU.MOREX.r3.3HG0304680 (ACO44685.1) and HORVU.MOREX.r3.4HG0392240 (CAA38036.1) see Fig. 1; Table 1 and supplementary Table 2. Furthermore, these previous genes encoding Plastid glutamine synthetase 2 [*T. Aestivum*], Glutamine synthetase leaf isozyme, Chloroplast, Cytosolic heat shock protein 90 [*H. vulgare*], predicted protein (*H. vulgare*), thioredoxin peroxidase (*H. vulgare*), Elongation factor Tu, chloroplast (*A. thaliana*), cysteine-rich receptor-like protein kinase 10 (*R. communis*), isocitrate dehydrogenase (*H. vulgare*), Beta-amylase, 70 kDa heat shock protein (H. vulgare), glycine rich protein, RNA binding protein (*H. vulgare*), UDPglucose 6-dehydrogenase (*Zea mays*), L-ascorbate peroxidase (*H. vulgare*) glycosyltransferase 75 (*T. aestivum*), reversibly glycosylated polypeptide (*T. aestivum*), Leucine-rich repeat family protein LRR (*A. thaliana*), Alcohol dehydrogenase I (*O. eichingeri*), fructose-bisphosphate aldolase (*H. vulgare*) and Heat shock protein 21, chloroplast see Fig. 2; Table 1 and supplementary Table S2.

### Predication the putative expression pattern of our target genes at various tissue, whole spike and provascular tissue based on*H. vulgare*transcript expression

**Fig. 3:**
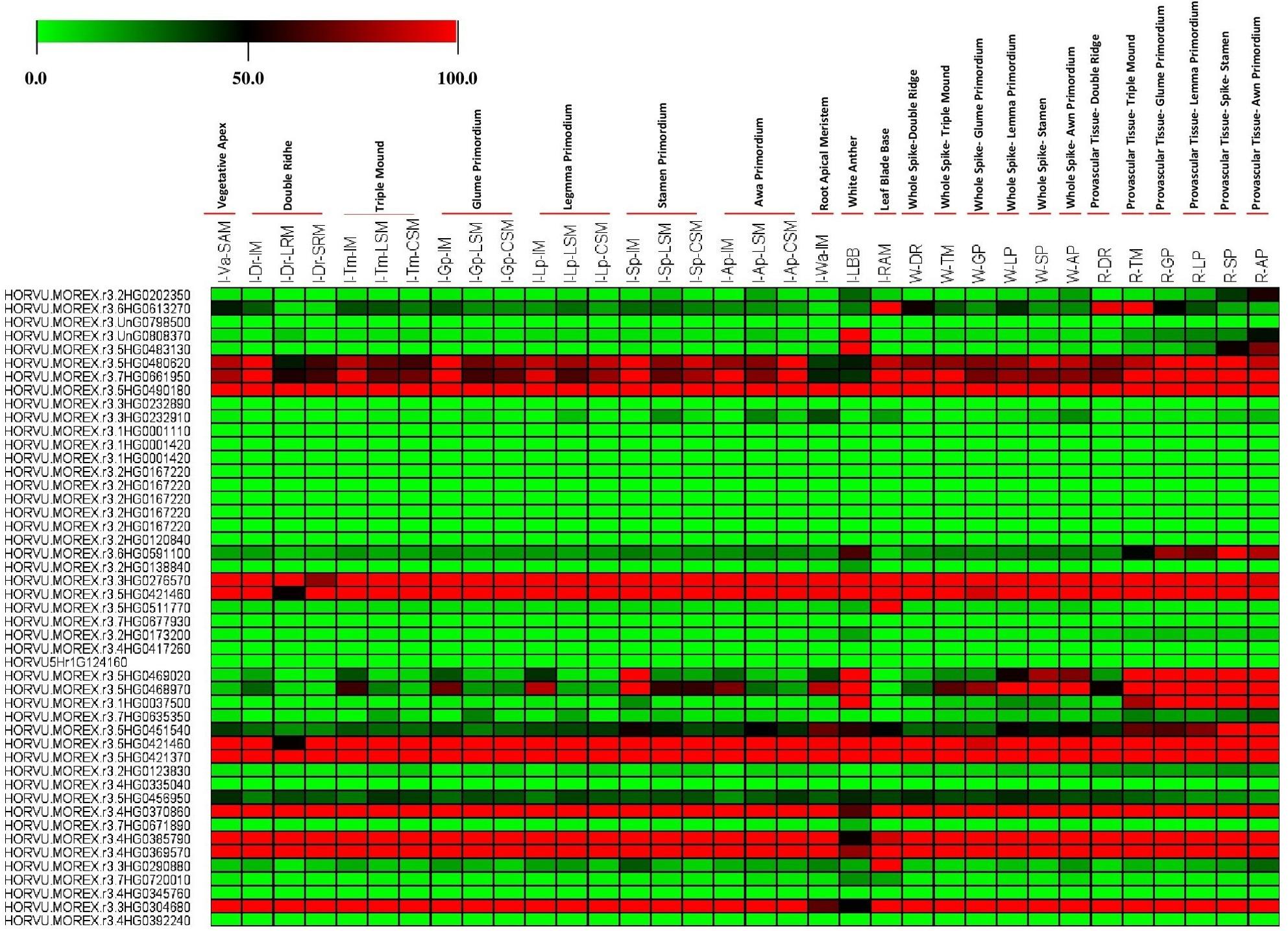
Heat map represent the putative expression pattern of our target genes at various tissue, whole spike and provascular tissue based on *H. vulgare* transcript expression. The color represents the expression scale (the more intense the red color, the more gene expression). Multi Experiment Viewer (MeV_4_9_0) software was used to depict transcript levels

The putative tissue expression of our candidate genes from *H. vulgare* at various tissue, whole spike and provascular tissue based on *H. vulgare* transcript expression was analyzed to realize their roles and functions in drought tolerance and other a biotic stress Fig. 3. The results showed that some of our target genes from *H. vulgare* were abundantly expressed across all tissues at various tissue, whole spike and provascular tissue. For example, the HORVU.MOREX.r3.5HG0480620 (gi|357158586), HORVU.MOREX.r3.7HG0661950 (gi|51090748), HORVU.MOREX.r3.5HG0490180 (gi|32765549), HORVU.MOREX.r3.3HG0276570 (gi|326493350), HORVU.MOREX.r3.5HG0421460 (gi|326493798), HORVU.MOREX.r3.5HG0421460 (gi|326493798), HORVU.MOREX.r3.5HG0421370 (gi|326493636), HORVU.MOREX.r3.4HG0370860 (AAC32060.1), HORVU.MOREX.r3.4HG0385790 (CAA06996.1), HORVU.MOREX.r3.4HG0369570 (ADK56176.1) and HORVU.MOREX.r3.3HG0304680 (ACO44685.1) that is connected to The following are predicted: cytosolic heat shock protein 90 [*H. vulgare*], putative chaperonin 21 precursor [*O. sativa* Japonica], 20 kDa chaperonin, chloroplastic-like (*Brachypodium distachyon*), isocitrate dehydrogenase (*H. vulgare*), glycine rich protein, RNA binding protein (*H. vulgare*), glycine rich protein, RNA binding protein (*H. vulgare*), enolase (*H. vulgare*), 20 S proteasome subunit PAE1 (*A. thaliana*), L-ascorbate peroxidase (*H. vulgare*), glycosyltransferase 75 (*T. aestivum*) and fructose-bisphosphate aldolase (*H. vulgare*) see Fig. 3; Table 1 and supplementary Table S3.

### Analyses of different expression pattern of target genes at Putative expression pattern of our target genes at various tissues under light and shade based on *H. vulgare*transcript expression

**Fig. 4:**
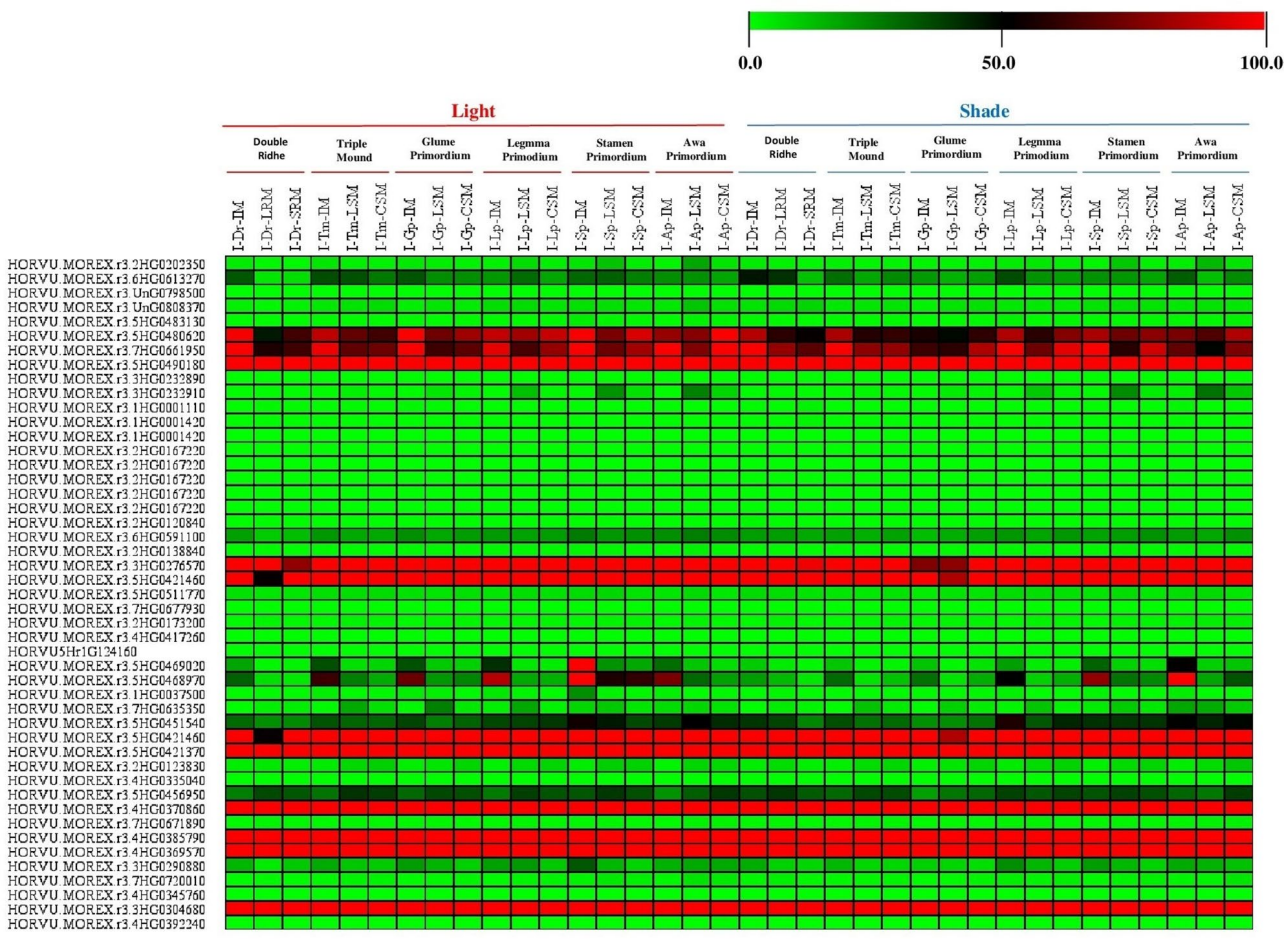
Heat map represent the Putative expression pattern of our target genes at various tissues under light and shade based on *H. vulgare* transcript expression. The color represents the expression scale (the more intense the red color, the more gene expression: Multi Experiment Viewer (MeV_4_9_0) software was used to depict transcript levels

The putative tissue expression of our candidate genes from *H. vulgare* under light and shade based on *H. vulgare* transcript expression was analyzed to comprehend their operations and involvement in a biotic stress and drought tolerance. Figure 4 some of our target genes from H. vulgare were found to be substantially expressed in all tissues, according to the results. For example, the HORVU.MOREX.r3.7HG0661950 (gi|51090748), HORVU.MOREX.r3.5HG0490180 (gi|32765549), HORVU.MOREX.r3.3HG0232890 (gi|326516152), HORVU.MOREX.r3.3HG0276570 (gi|326493350), HORVU.MOREX.r3.5HG0421460 (gi|326493798), HORVU.MOREX.r3.5HG0421460 (gi|326493798), HORVU.MOREX.r3.5HG0421370 (gi|326493636), HORVU.MOREX.r3.4HG0370860 (AAC32060.1), HORVU.MOREX.r3.4HG0385790 (CAA06996.1), HORVU.MOREX.r3.4HG0369570 (ADK56176.1) and HORVU.MOREX.r3.3HG0304680 (ACO44685.1) that associated with putative chaperonnin 21 precursor [*O. sativa*], Cytosolic heat shock protein 90 [*H. vulgare*], predicted protein (*H. vulgare*), isocitrate dehydrogenase (*H. vulgare*), glycine rich protein, RNA binding protein (*H. vulgare*), glycine rich protein, enolase (*H. vulgare*), 20 S proteasome subunit PAE1 (*A. thaliana*), L-ascorbate peroxidase (*H. vulgare*), glycosyltransferase 75 (*T. aestivum*) and fructose-bisphosphate aldolase (*H. vulgare*) see Fig. 4; Table 1 and supplementary Table S4.

## Discussion

Drought and other stresses co-occur in the natural environment, especially in dry hot areas [33, 34]. An essential and top research objective is learning more about how plants react to drought and salinity stresses [35–37]. Moreover, in our study we used the 2-DE gels for proteome analysis and determine the key proteins linked to stress and their related genes, to comprehend the fundamental processes behind barley’s resistance to drought stress. Based on our results we identified among 56 proteins and most of them are related to the drought and other stresses tolerance in this study see Table 1. These previous proteins were up-regulated or down-regulated or remained unaltered in barely plant under drought stress in compared with control see Figure. 1 Proteins whose quantity is highly dependent on drought and other stresses are primarily linked to a number of biological processes, including photosynthesis, plant growth and development [38].

As results, Fig. 1A and B display two distinct 2D-GE pictures, and after analyzing each gel we found unique spots for everyone and common spots between the two gels. Also, Fig. 1A and B displays the correlation between the spot sizes and the expression of proteins under the stress and control condition. In particular, the gel analysis for leaf sample under 10% PEG has 10 unique spots such as; spot 13 (HORVU1Hr1G001420), spot 17 (HORVU2Hr1G073760), spot 24 (HORVU2Hr1G026810), spot 29 (HORVU5Hr1G096370), spot 34 (HORVU5Hr1G038630), spot 42 (HORVU2Hr1G029840), spot 44 (HORVU4Hr1G040770), spot 49 (HORVU3Hr1G073780), spot 50 (HORVU7Hr1G090240) and spot 56 (HORVU4Hr1G063350), and these previous spots related with various proteins such as; LMW- glutenin, 2-cys peroxiredoxin BAS1 (*T. aestivum*), Thioredoxin peroxidase (*H. vulgare*), UDP-glucose 6-dehydrogenase (*Zea mays*), Ribulose-bisphosphate carboxylase small chain, Putative hydrolase (*A. thaliana*), Proteasome subunit alpha type-5-A (*A.thaliana*), Reversibly glycosylated polypeptide (*T. aestivum*), Leucine-rich repeat family protein LRR (*A. thaliana*) and heat shock protein 21, chloroplast see Fig. 1; Table 1 and supplementary Table 1. And from our data analysis we found these previous proteins were classified and showed various biological functions in barley leaf see Table 1 [6]. they found positively relationship between the relative water content in leave and the total Rubisco activity, which mean the Ribulose-bisphosphate carboxylase small chain can plays a complex role under drought conditions, through maintain photosynthetic rates under stress and these response based on the kind, intensity, and length of the stress. Also, Ayub et al., 2011 and Ding et al., 2024 [11, 30, 31, 39], they found epistatic relationship between the light and dark from side and the leaf carbon metabolism, photosynthesis and respiration under drought stress from the other side. Any changes in sustained drought levels are followed by changes in Rubisco enzyme activity [11, 30, 31, 39]. Moreover, as we know the barley and some other cereal crops quality are governed largely by two types of glutenin proteins: the high molecular weight (HMW) and the low molecular weight (LMW) - glutenin subunit [40]. Moreover [41–43], study the influence of heat and drought stress on the levels of various glutenin types, and found a linear relationship between the impact of drought stress and the LMW-glutenin, and any alter in the composition and properties of glutenin may be affected on strength and bread-making quality. For Thioredoxin peroxidase protein, several studies have been reported about its vital function in the growth and development of a plant under various abiotic Stress as a component of a redox system, and it is ability to modulate the redox signaling during plant development, growth and stress adaptation through dithiol-disulfide exchanges [44–46]. And this dithioldisulfide is essential for both signal transduction and redox sensing pathways [46]. And another proteins such as; Heat shock protein 21 and 2-cys peroxiredoxin BAS1 (*T. aestivum*) have been reported as a unique spot under 10% PEG, and these two proteins plays a significant part in plants’ ability to withstand drought stress. Through activation the antioxidant enzymatic system, this works on avoiding and scavenging reactive oxygen species (ROS) over-accumulation by 2-cys peroxiredoxin enzyme [10, 11, 47]. Heat shock protein 21 reduces the effect of drought stress by mediates stress signal transduction, controlling with ATPase-coupled, and interactions with co-chaperone proteins [11, 48]. Furthermore, the function of UDP-glucose 6-dehydrogenase (UGDH) in tolerance to drought stress has been reported by [49, 50]. They found the UGDH can enhance the plant response to drought stress through their effect on cell wall potentially and composition. And the UGDH enzyme have ability to conversion of UDP-glucose to UDP-glucuronic acid by catalyze response, and the second component is a precursor for different cell wall polysaccharides, such as; hemicellulose and pectin. For example, the spot 32 (HORVU4Hr1G089510) was related to Beta-amylase (BAM) protein and this protein plays an important function in plants drought stress tolerance through degradation the starch into maltose sugar, and this last one can used by plant as source of energy and as an osmoprotectant to protect plant from plants cope [51–53]. Also, the spot 47 (HORVU4Hr1G057210) was related to L-ascorbate peroxidase and this enzyme helping plants cope with drought stress through utilizes ascorbate (vitamin C) as a substrate to reduce H2O2 to water, and decrease the oxidative damage imposed by drought stress. By scavenge ROS then mitigating the harmful of the effects of ROS produced [11, 39, 54–56]. Moreover, the spot 51 (HORVU4Hr1G016810) was involved in Alcohol dehydrogenase I (ADH1), and this enzyme has a vital function in drought and other stress tolerance by catalyzing the conversion of acetaldehyde to ethanol, and this helps plant under various stress to regenerate NAD + for continue energy production under anaerobic conditions that occur under drought [57]– [58]. At the end, the spot 52 (HORVU3Hr1G088540) was related with Fructose-bisphosphate aldolase enzyme (FBA), and this enzyme play an important role in drought and other abiotic stress tolerance by converting fructose-1,6-bisphosphate into dihydroxyacetone phosphate and glyceraldehyde-3-phosphate in the Calvin cycle of photosynthesis pathway for producing sugars that fuel plant growth and metabolism see Table 1 [59–62].

On the other hand, numerous investigations have revealed that the effects of drought and other abiotic stress not ceasing only at the leave of plantlet but they can effects on various tissues at transverse and sagittal sections, various tissues under light and shade, and various tissues at whole spike and provascular tissue. For that, in this study we used various tools and parameters from the barley Plant Fluorescent Electronic Pictograph Browsers (barley eFP browsers) (http://bar.utoronto.ca/eplant_Arabidopsis/) for predicting the putative expression of our target genes at all previous tissues see (Figs. 2, 3 and 4). And from our data analysis, we found all our genes have different expression levels at each tissue and under various development stages and growth conditions. In addition, some genes have variable putative expression from tissue to another one under various development stages and conditions see (Figs. 2, 3 and 4).

## Conclusion

Our study assessed the impact of drought stress on the levels of expression of different proteins in barley seedling. Moreover, in this study we used two-dimensional electrophoresis (2D-gel), mass spectrometry and bioinformatics software to investigate barley seedling proteins’ composition and function under drought stress in comparison with control. Our results showed about 56 spots (proteins) from various unique and common proteins, which are related with the ability of barley plants to tolerance the drought stress. And these proteins have various biological functions and play an important role in drought and other abiotic stress tolerance such as; spot 32 (HORVU4Hr1G089510), spot 47 (HORVU4Hr1G057210) and spot 51 (HORVU4Hr1G016810) which related with L-ascorbate peroxidase enzyme, Alcohol dehydrogenase I enzyme and Fructose-bisphosphate aldolase enzyme, respectively. In addition, the putative expression patterns of the identified proteins at various tissues under different conditions by BAR databases as bioinformatics tools were also completed. At the end, this information can be relied upon in future programs related to the production of new barley genotypes that are tolerant to drought and other biotic stresses.

## Supplementary Information

Supplementary Material 1. Supplementary Figure S1: The two-dimensional gel electrophoresis (2-DE) before and after editing. (A) gel of protein before editing at normal condition (control). (B) gel of protein before editing at 10% drought condition. (C) gel of protein after editing at normal condition (control) . (D) gel of protein after editing at 10% drought condition.

Supplementary Material 2. Supplementary Table S1: List of spots proteins and their putative function and form of each spot from the 2-DE gel.

Supplementary Material 3. Supplementary Table S2: The putative expression of our candidate genes at Transverse Section and Sagital Sextion. Supplementary Table S3: The putative expression of our candidate genes at different tissue types. Supplementary Table S4: The putative expression of our candidate genes at different tissue types under light and shade conditions.

## Data Availability

Data is provided within the manscript or supplementary information files.
