# Supplementary material for: Proteomic analysis of proteins responsive to drought stress in barley
Source: BMC Plant Biol. 2026 Feb 11;26:385. doi: 10.1186/s12870-026-08176-8 (PMC12930857; doi:10.1186/s12870-026-08176-8)
Supplement: Supplementary file 1 — Supplementary Material 1. Supplementary Figure S1: The two-dimensional gel electrophoresis (2-DE) before and after editing. (A) gel of protein before editing at normal condition (control). (B) gel of protein before editing at 10% drought condition. (C) gel of protein after editing at normal condition (control) . (D) gel of protein after editing at 10% drought condition. [file 12870_2026_8176_MOESM1_ESM.pptx]

## Slide 1
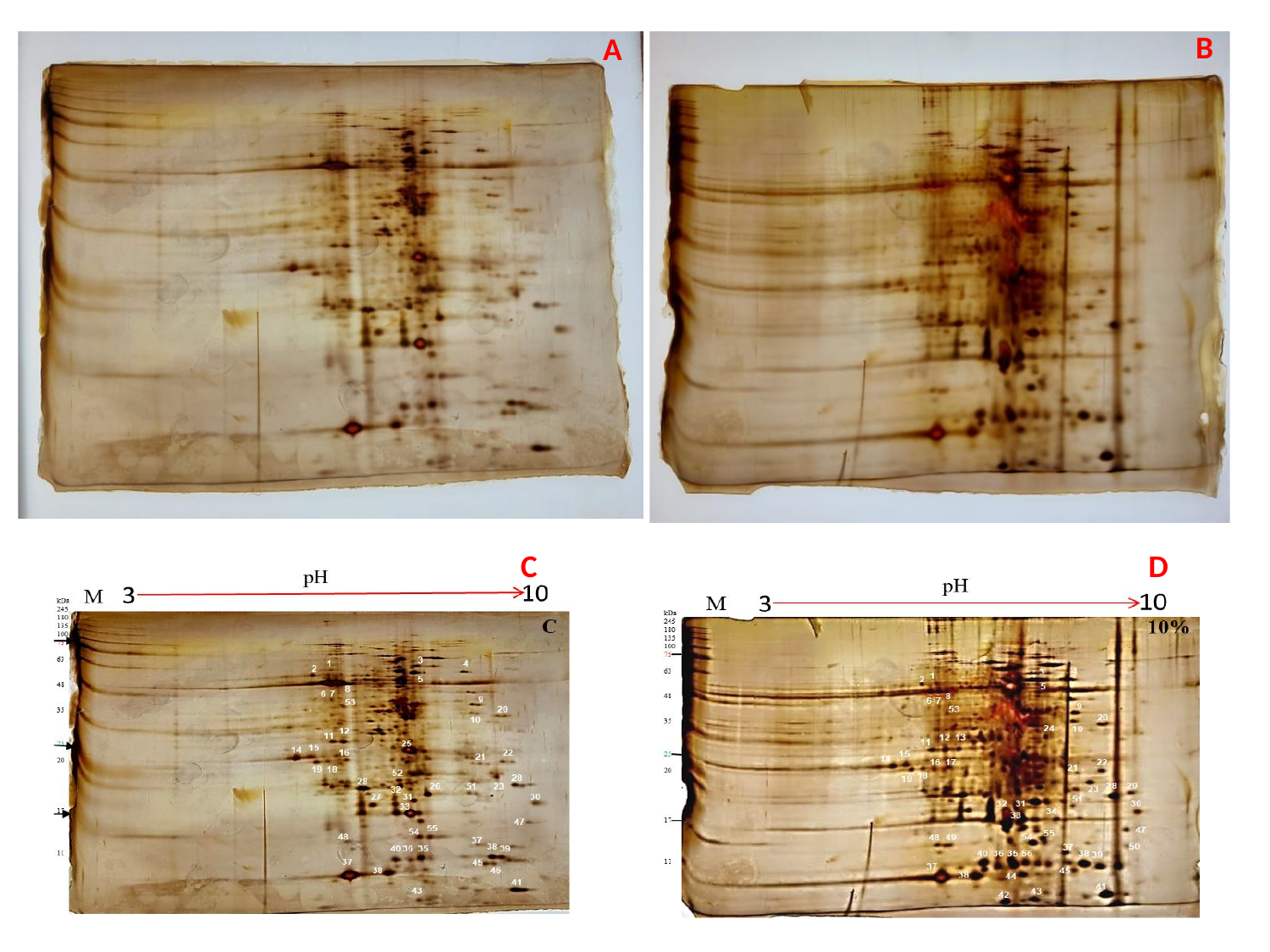

B
A
C
D

## Slide 2
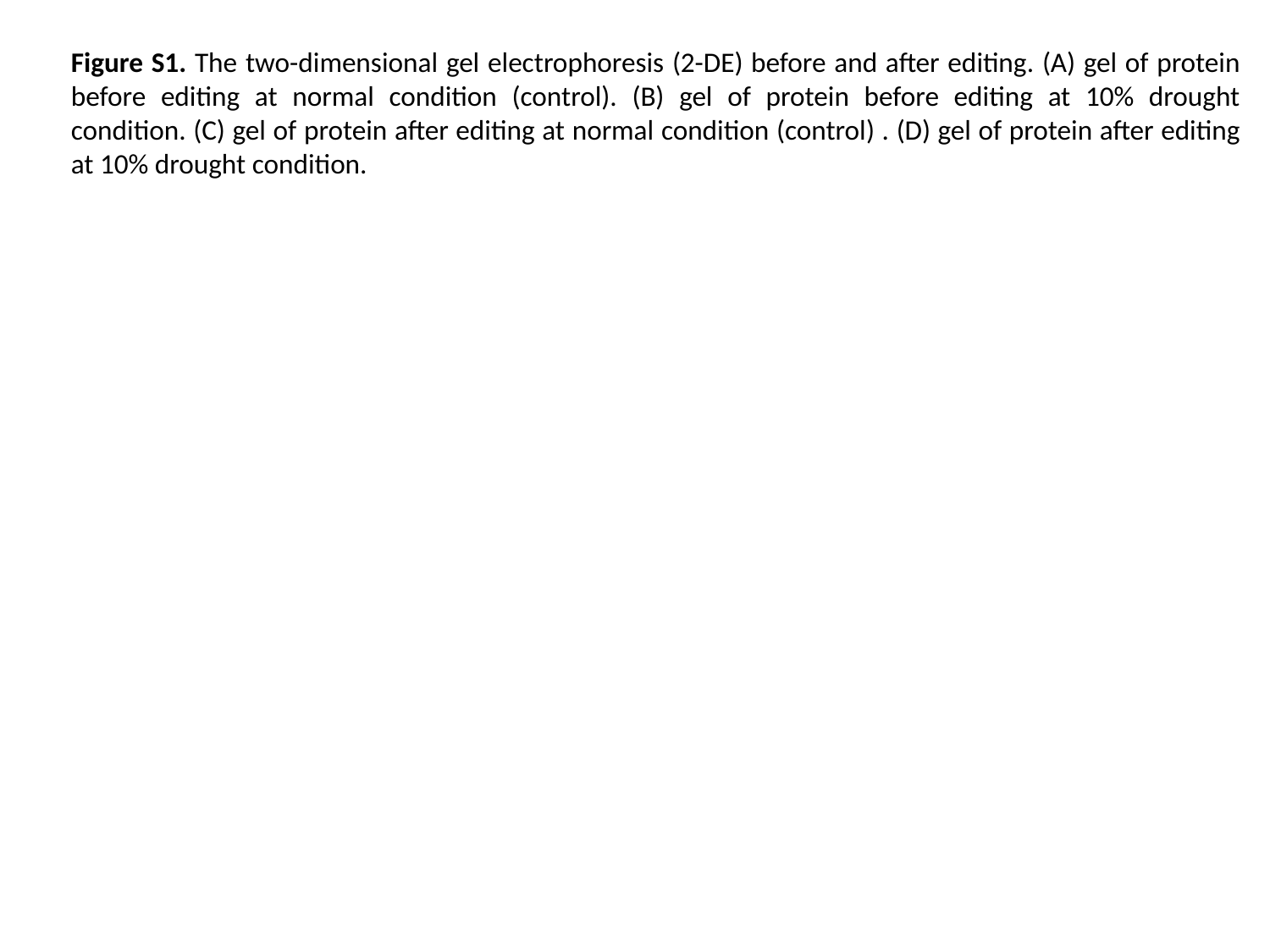

Figure S1. The two-dimensional gel electrophoresis (2-DE) before and after editing. (A) gel of protein before editing at normal condition (control). (B) gel of protein before editing at 10% drought condition. (C) gel of protein after editing at normal condition (control) . (D) gel of protein after editing at 10% drought condition.

## Slide 3
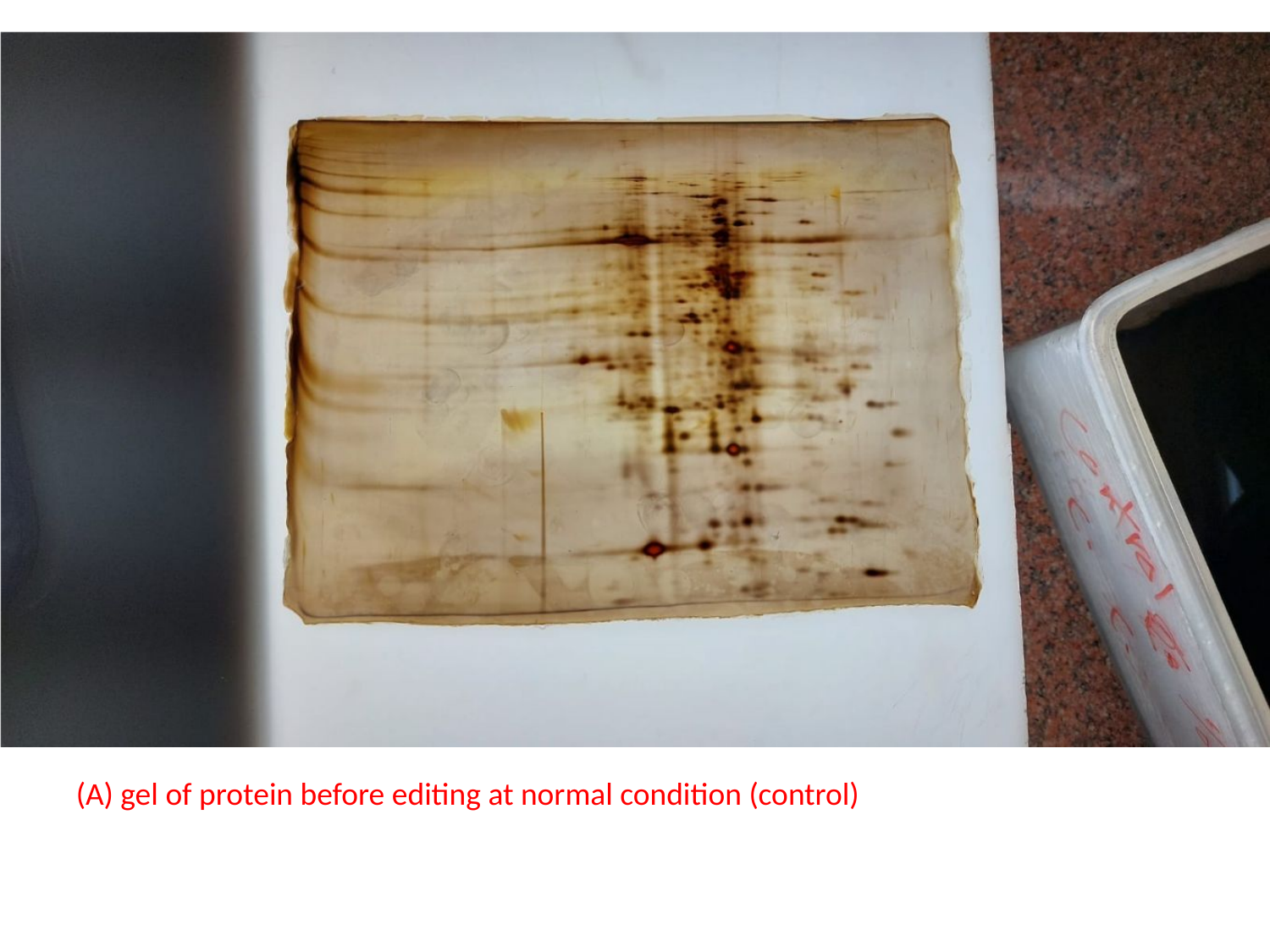

(A) gel of protein before editing at normal condition (control)

## Slide 4
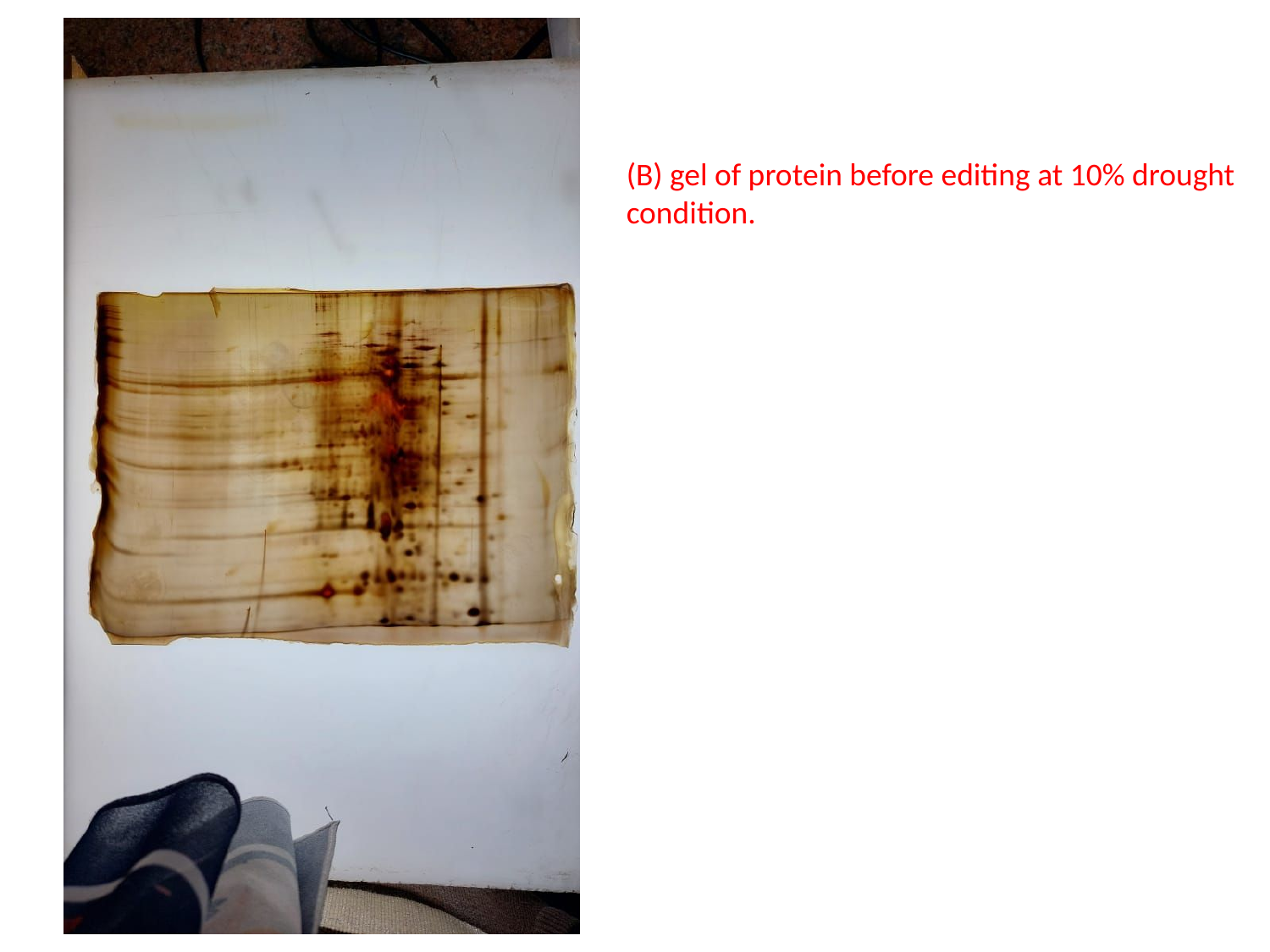

(B) gel of protein before editing at 10% drought condition.
