# Supplementary material for: Proteomic analysis of proteins responsive to drought stress in barley
Source: BMC Plant Biol. 2026 Feb 11;26:385. doi: 10.1186/s12870-026-08176-8 (PMC12930857; doi:10.1186/s12870-026-08176-8)
Supplement: Supplementary file 2 — Supplementary Material 2. Supplementary Table S1: List of spots proteins and their putative function and form of each spot from the 2-DE gel. [file 12870_2026_8176_MOESM2_ESM.pptx]

## Slide 1
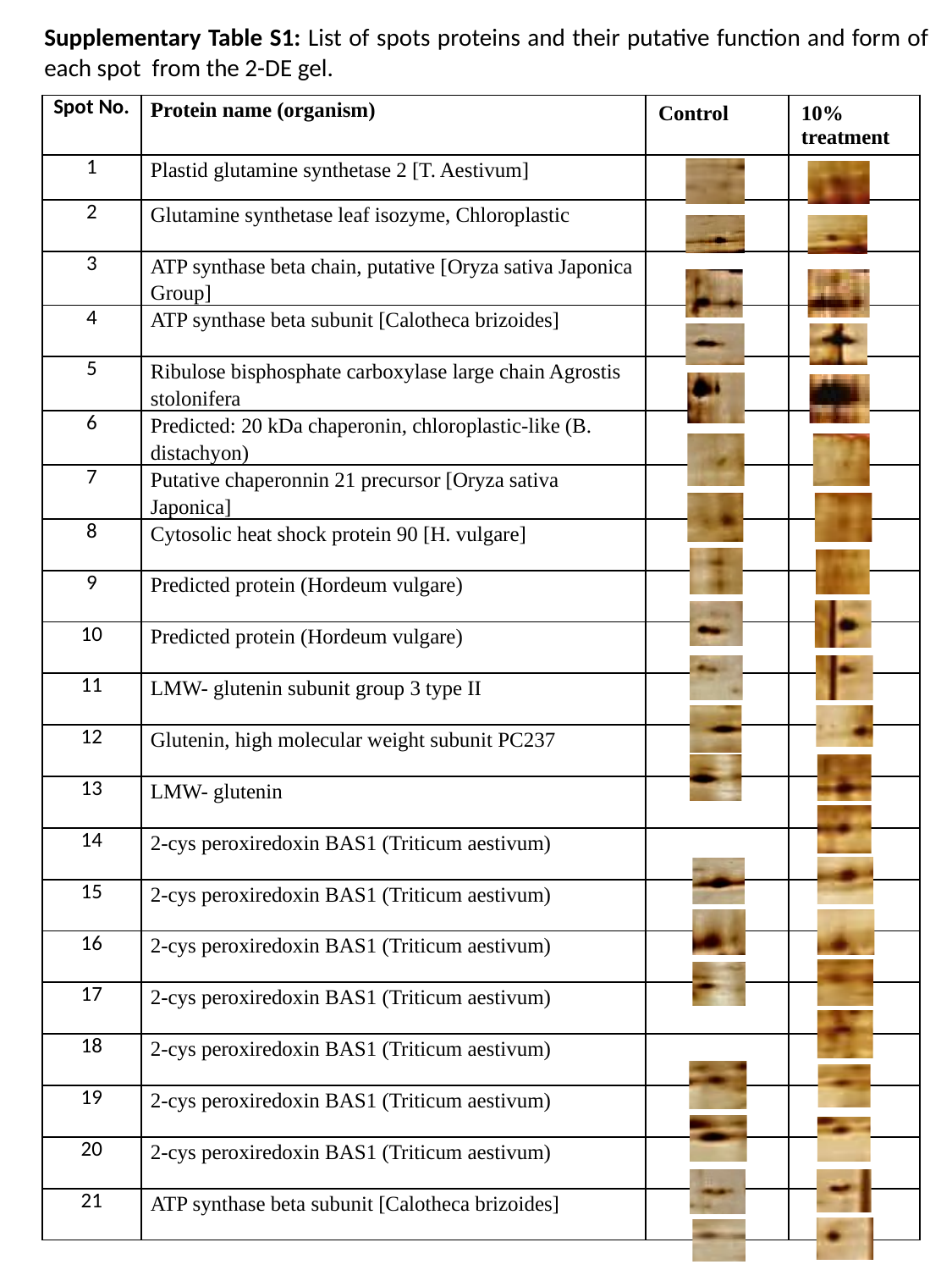

Supplementary Table S1: List of spots proteins and their putative function and form of each spot from the 2-DE gel.
| Spot No. | Protein name (organism) | Control | 10% treatment |
| --- | --- | --- | --- |
| 1 | Plastid glutamine synthetase 2 [T. Aestivum] | | |
| 2 | Glutamine synthetase leaf isozyme, Chloroplastic | | |
| 3 | ATP synthase beta chain, putative [Oryza sativa Japonica Group] | | |
| 4 | ATP synthase beta subunit [Calotheca brizoides] | | |
| 5 | Ribulose bisphosphate carboxylase large chain Agrostis stolonifera | | |
| 6 | Predicted: 20 kDa chaperonin, chloroplastic-like (B. distachyon) | | |
| 7 | Putative chaperonnin 21 precursor [Oryza sativa Japonica] | | |
| 8 | Cytosolic heat shock protein 90 [H. vulgare] | | |
| 9 | Predicted protein (Hordeum vulgare) | | |
| 10 | Predicted protein (Hordeum vulgare) | | |
| 11 | LMW- glutenin subunit group 3 type II | | |
| 12 | Glutenin, high molecular weight subunit PC237 | | |
| 13 | LMW- glutenin | -- | |
| 14 | 2-cys peroxiredoxin BAS1 (Triticum aestivum) | | |
| 15 | 2-cys peroxiredoxin BAS1 (Triticum aestivum) | | |
| 16 | 2-cys peroxiredoxin BAS1 (Triticum aestivum) | | |
| 17 | 2-cys peroxiredoxin BAS1 (Triticum aestivum) | -- | |
| 18 | 2-cys peroxiredoxin BAS1 (Triticum aestivum) | | |
| 19 | 2-cys peroxiredoxin BAS1 (Triticum aestivum) | | |
| 20 | 2-cys peroxiredoxin BAS1 (Triticum aestivum) | | |
| 21 | ATP synthase beta subunit [Calotheca brizoides] | | |

## Slide 2
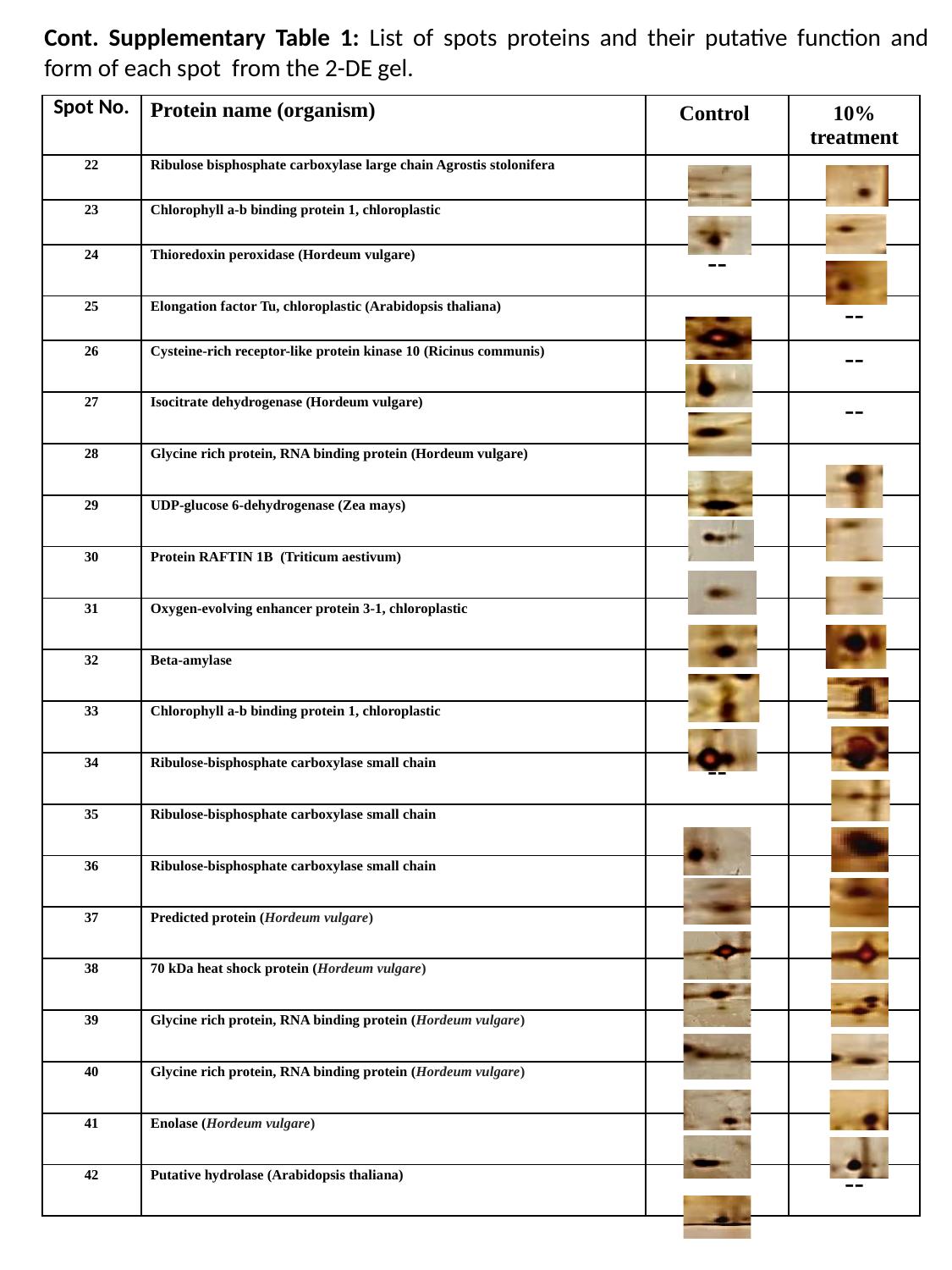

Cont. Supplementary Table 1: List of spots proteins and their putative function and form of each spot from the 2-DE gel.
| Spot No. | Protein name (organism) | Control | 10% treatment |
| --- | --- | --- | --- |
| 22 | Ribulose bisphosphate carboxylase large chain Agrostis stolonifera | | |
| 23 | Chlorophyll a-b binding protein 1, chloroplastic | | |
| 24 | Thioredoxin peroxidase (Hordeum vulgare) | -- | |
| 25 | Elongation factor Tu, chloroplastic (Arabidopsis thaliana) | | -- |
| 26 | Cysteine-rich receptor-like protein kinase 10 (Ricinus communis) | | -- |
| 27 | Isocitrate dehydrogenase (Hordeum vulgare) | | -- |
| 28 | Glycine rich protein, RNA binding protein (Hordeum vulgare) | | |
| 29 | UDP-glucose 6-dehydrogenase (Zea mays) | | |
| 30 | Protein RAFTIN 1B (Triticum aestivum) | | |
| 31 | Oxygen-evolving enhancer protein 3-1, chloroplastic | | |
| 32 | Beta-amylase | | |
| 33 | Chlorophyll a-b binding protein 1, chloroplastic | | |
| 34 | Ribulose-bisphosphate carboxylase small chain | -- | |
| 35 | Ribulose-bisphosphate carboxylase small chain | | |
| 36 | Ribulose-bisphosphate carboxylase small chain | | |
| 37 | Predicted protein (Hordeum vulgare) | | |
| 38 | 70 kDa heat shock protein (Hordeum vulgare) | | |
| 39 | Glycine rich protein, RNA binding protein (Hordeum vulgare) | | |
| 40 | Glycine rich protein, RNA binding protein (Hordeum vulgare) | | |
| 41 | Enolase (Hordeum vulgare) | | |
| 42 | Putative hydrolase (Arabidopsis thaliana) | | -- |

## Slide 3
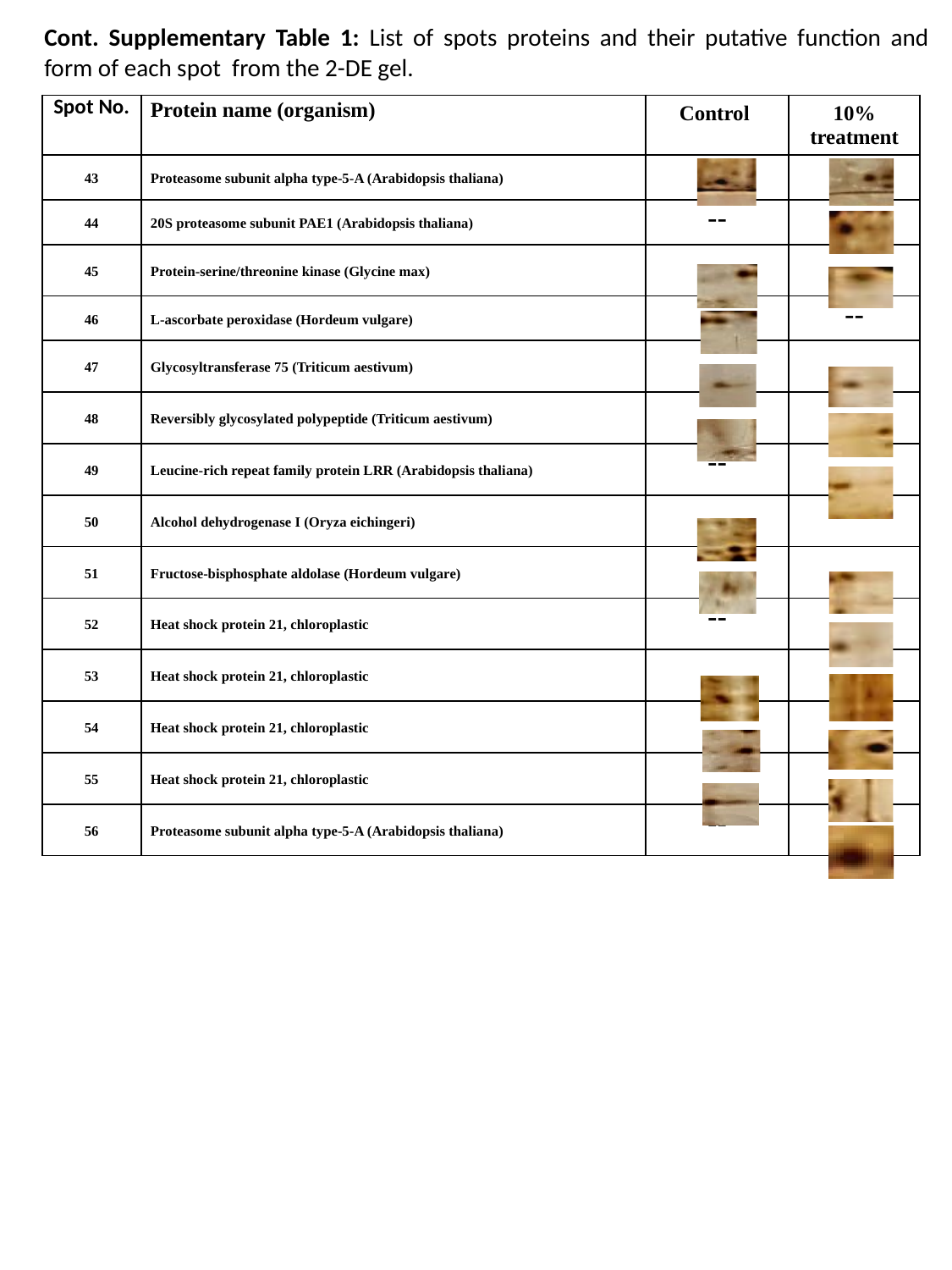

Cont. Supplementary Table 1: List of spots proteins and their putative function and form of each spot from the 2-DE gel.
| Spot No. | Protein name (organism) | Control | 10% treatment |
| --- | --- | --- | --- |
| 43 | Proteasome subunit alpha type-5-A (Arabidopsis thaliana) | | |
| 44 | 20S proteasome subunit PAE1 (Arabidopsis thaliana) | -- | |
| 45 | Protein-serine/threonine kinase (Glycine max) | | |
| 46 | L-ascorbate peroxidase (Hordeum vulgare) | | -- |
| 47 | Glycosyltransferase 75 (Triticum aestivum) | | |
| 48 | Reversibly glycosylated polypeptide (Triticum aestivum) | | |
| 49 | Leucine-rich repeat family protein LRR (Arabidopsis thaliana) | -- | |
| 50 | Alcohol dehydrogenase I (Oryza eichingeri) | | -- |
| 51 | Fructose-bisphosphate aldolase (Hordeum vulgare) | | |
| 52 | Heat shock protein 21, chloroplastic | -- | |
| 53 | Heat shock protein 21, chloroplastic | | |
| 54 | Heat shock protein 21, chloroplastic | | |
| 55 | Heat shock protein 21, chloroplastic | | |
| 56 | Proteasome subunit alpha type-5-A (Arabidopsis thaliana) | -- | |
